# Development and validation of a risk prediction model for invasiveness of pure ground-glass nodules based on a systematic review and meta-analysis

**DOI:** 10.1186/s12880-024-01313-5

**Published:** 2024-06-17

**Authors:** Yantao Yang, Libin Zhang, Han Wang, Jie Zhao, Jun Liu, Yun Chen, Jiagui Lu, Yaowu Duan, Huilian Hu, Hao Peng, Lianhua Ye

**Affiliations:** 1 Department of Thoracic and Cardiovascular Surgery, Yunnan Cancer Hospital, The Third Affiliated Hospital of Kunming Medical University, No. 519 Kunzhou Road, Xishan District, Kunming, China; 2 Department of Thoracic Surgery, The First People’s Hospital Of Yunnan Province, Kunming City, Yunnan Province China

**Keywords:** Pure ground glass nodules(pGGNs), Prediction model, Lung adenocarcinoma, Radiologic characteristic, Invasiveness

## Abstract

**Background:**

Assessing the aggressiveness of pure ground glass nodules early on significantly aids in making informed clinical decisions.

**Objective:**

Developing a predictive model to assess the aggressiveness of pure ground glass nodules in lung adenocarcinoma is the study’s goal.

**Methods:**

A comprehensive search for studies on the relationship between computed tomography(CT) characteristics and the aggressiveness of pure ground glass nodules was conducted using databases such as PubMed, Embase, Web of Science, Cochrane Library, Scopus, Wanfang, CNKI, VIP, and CBM, up to December 20, 2023. Two independent researchers were responsible for screening literature, extracting data, and assessing the quality of the studies. Meta-analysis was performed using Stata 16.0, with the training data derived from this analysis. To identify publication bias, Funnel plots and Egger tests and Begg test were employed. This meta-analysis facilitated the creation of a risk prediction model for invasive adenocarcinoma in pure ground glass nodules. Data on clinical presentation and CT imaging features of patients treated surgically for these nodules at the Third Affiliated Hospital of Kunming Medical University, from September 2020 to September 2023, were compiled and scrutinized using specific inclusion and exclusion criteria. The model’s effectiveness for predicting invasive adenocarcinoma risk in pure ground glass nodules was validated using ROC curves, calibration curves, and decision analysis curves.

**Results:**

In this analysis, 17 studies were incorporated. Key variables included in the model were the largest diameter of the lesion, average CT value, presence of pleural traction, and spiculation. The derived formula from the meta-analysis was: 1.16×the largest lesion diameter + 0.01 × the average CT value + 0.66 × pleural traction + 0.44 × spiculation. This model underwent validation using an external set of 512 pure ground glass nodules, demonstrating good diagnostic performance with an ROC curve area of 0.880 (95% CI: 0.852–0.909). The calibration curve indicated accurate predictions, and the decision analysis curve suggested high clinical applicability of the model.

**Conclusion:**

We established a predictive model for determining the invasiveness of pure ground-glass nodules, incorporating four key radiological indicators. This model is both straightforward and effective for identifying patients with a high likelihood of invasive adenocarcinoma.

**Supplementary Information:**

The online version contains supplementary material available at 10.1186/s12880-024-01313-5.

## Introduction

Recent advancements in lung cancer screening have led to the detection of an increasing number of early lung adenocarcinomas, often indicated by ground glass nodules(GGNs) [1]. In 2021, the World Health Organization categorized lung adenocarcinomas into three stages: adenocarcinoma in situ(AIS), minimally invasive adenocarcinoma(MIA), and invasive adenocarcinoma(IAC), based on their progression [2]. AIS and MIA are called preinvasive lesions(PIL).GGNs are classified into pure ground glass nodules(pGGNs) and part solid nodules(PSNs) depending on the presence of solid components [3]. While pGGNs are typically perceived as less aggressive, with pathology usually corresponding to AIS and MIA, approximately 16-27% of these nodules still present with IAC pathology [4].

Complete resection typically results in a cure for patients with AIS and MIA. However, those with IAC face a recurrence risk even after complete resection [5, 6]. The approach to lymph node management also varies between these types. Lymph node treatment is not necessary for AIS and MIA, but lymph node dissection or sampling is essential for IAC [7]. Therefore, accurately identifying IAC is crucial in treating pGGNs.

Intraoperative freezing exhibits limitations in determining the invasiveness of pGGNs [8]. In comparison, imaging features offer more advantages in assessing the invasiveness of these nodules [9]. Several studies have indicated the effectiveness of imaging characteristics in identifying the invasiveness of pGGNs [10–12]. Given the variability in findings across different researchers, a meta-analysis was undertaken to examine the relationship between imaging features and the invasiveness of pure ground glass nodules. This analysis led to the development of a predictive model, aiming to guide clinical management of these nodules.

## Methods

### Study registration

The protocol was registered in the International PROSPERO with the registration number CRD42022362400.

### Study population characteristics

#### Derivation cohort

##### Participants

The patient data for the derivation cohort was sourced from a systematic review and meta-analysis. Two researchers independently explored a range of databases, such as PubMed, Embase, Web of Science, Cochrane Library, Scopus, Wanfang, CNKI, VIP, and CBM, up to December 20, 2023. They focused on finding relevant studies published in either Chinese or English. The search involved medical subject heading terms like computed tomography, ground-glass nodules, ground-glass opacities, adenocarcinoma in situ, micro-invasive adenocarcinoma, and invasive adenocarcinoma. Additionally, the bibliographies of chosen articles were reviewed to uncover more pertinent literature. In instances of disagreement, a third researcher was consulted to resolve the differences. Studies were included according to specific inclusion and exclusion criteria.Criteria for Inclusion: (1) Research exploring the link between computed tomography (CT) features and the invasiveness of pGGNs, published both domestically and internationally. (2) Utilization of surgery as the definitive method for pathological diagnosis. (3) Inclusion of both retrospective and prospective studies. (4) Studies providing raw data directly or through calculation. Criteria for Exclusion: (1) Exclusion of abstracts, reviews, and case reports. (2) Rejection of studies with duplicated data. (3) Disregarding studies with incomplete data or when specific data are unobtainable, even after author consultation. (4) Non-consideration of literature published in languages other than Chinese and English.

##### Data accessibility and quality evaluation

Initially, the evaluation of articles retrieved was conducted separately by two researchers, adhering to the established criteria for both inclusion and exclusion. This was followed by a comprehensive review together to ensure a consistent approach in their assessments. In instances of differing opinions, the lead author provided the decisive input, resolving these discrepancies through a collaborative dialogue involving all contributing authors. The methodology for data extraction from the selected papers involved key details such as the identification of the principal author, the publication year, the title of the study, the participant count, the maximum observed diameter, and a spectrum of characteristics observable in CT scans. These characteristics encompassed indicators like vascular convergence, the presence of spiculation, air bronchogram, vacuole sign, lobulation, the computed mean CT value, and signs of pleural traction. A separate, unbiased evaluation of each paper’s quality was carried out using the Newcastle-Ottawa Scale (NOS). This scale examines three critical domains: selection of the study (rated from 0 to 4 points), comparability among subjects (0 to 2 points), and the precision in assessing outcomes (0 to 3 points). Literature that achieved a NOS score of 5 or more was classified as high-caliber research.

#### Validation cohort

##### Participants

Our study evaluated 688 patients with pGGNs from the Third Affiliated Hospital of Kunming Medical University, admitted between September 2020 and September 2023. The patient selection followed specific inclusion and exclusion criteria. Inclusion criteria included: (1) Pre-surgical CT imaging data within two weeks showing one or more pGGNs; (2) Pathological diagnosis of lung adenocarcinoma (AIS, MIA, IAC) post-surgical resection of pGGNs; (3) Surgical intervention for one or more pGGNs; (4) No prior anti-tumor treatments like radiotherapy or chemotherapy; (5) Age 18 years or older. Exclusion criteria involved: (1) Patients with incomplete imaging data or medical records; (2) Lung infections that could affect image analysis; (3) Significant respiratory movement artifacts in images impairing imaging analysis; (4) Inconsistent locations of GGNs in postoperative pathology reports and preoperative CT images.

##### CT acquisition and image analysis

**Fig. 1:**
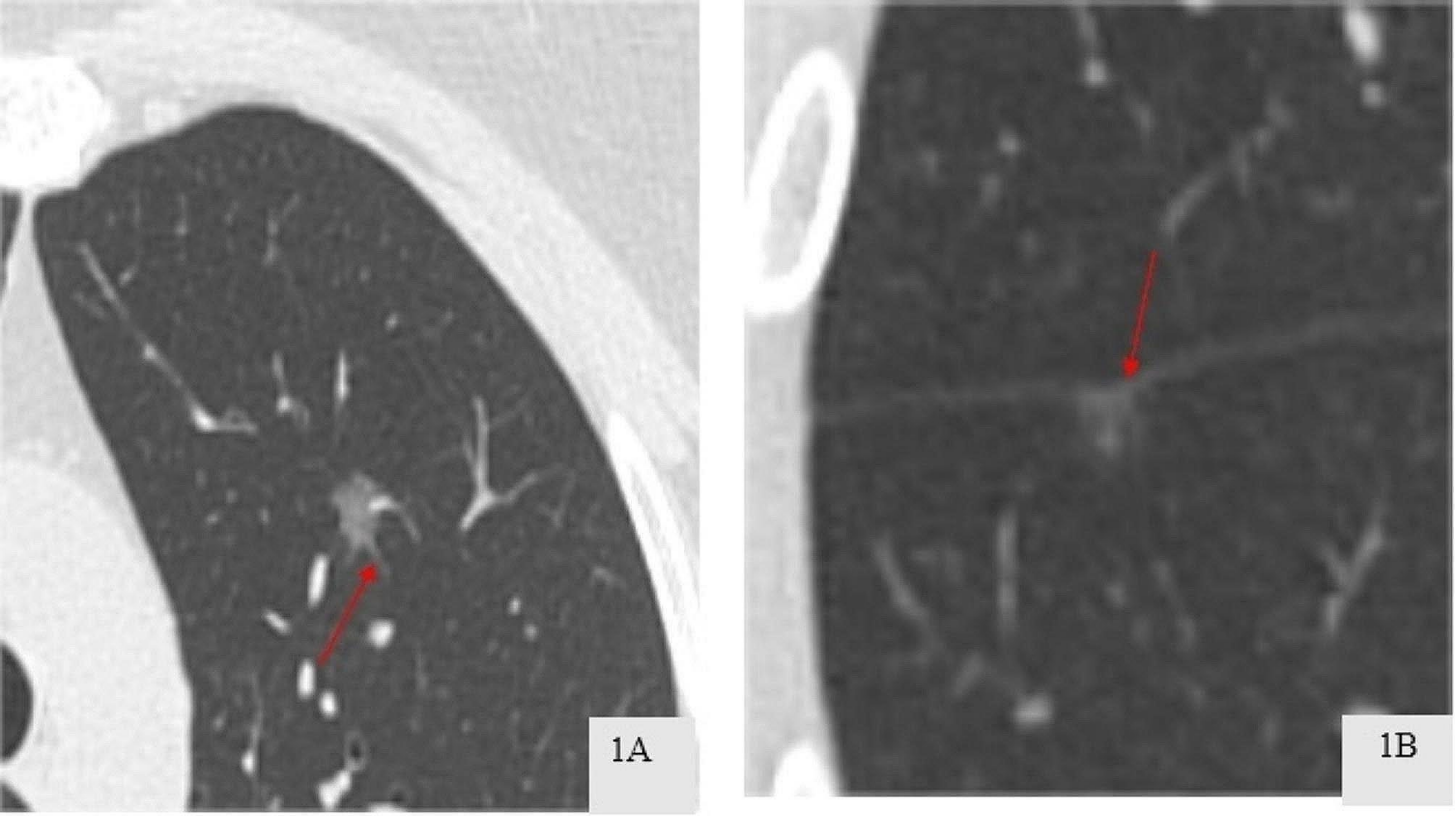
**A** Spiculation sign; **B** Pleura traction sign

**Fig. 2:**
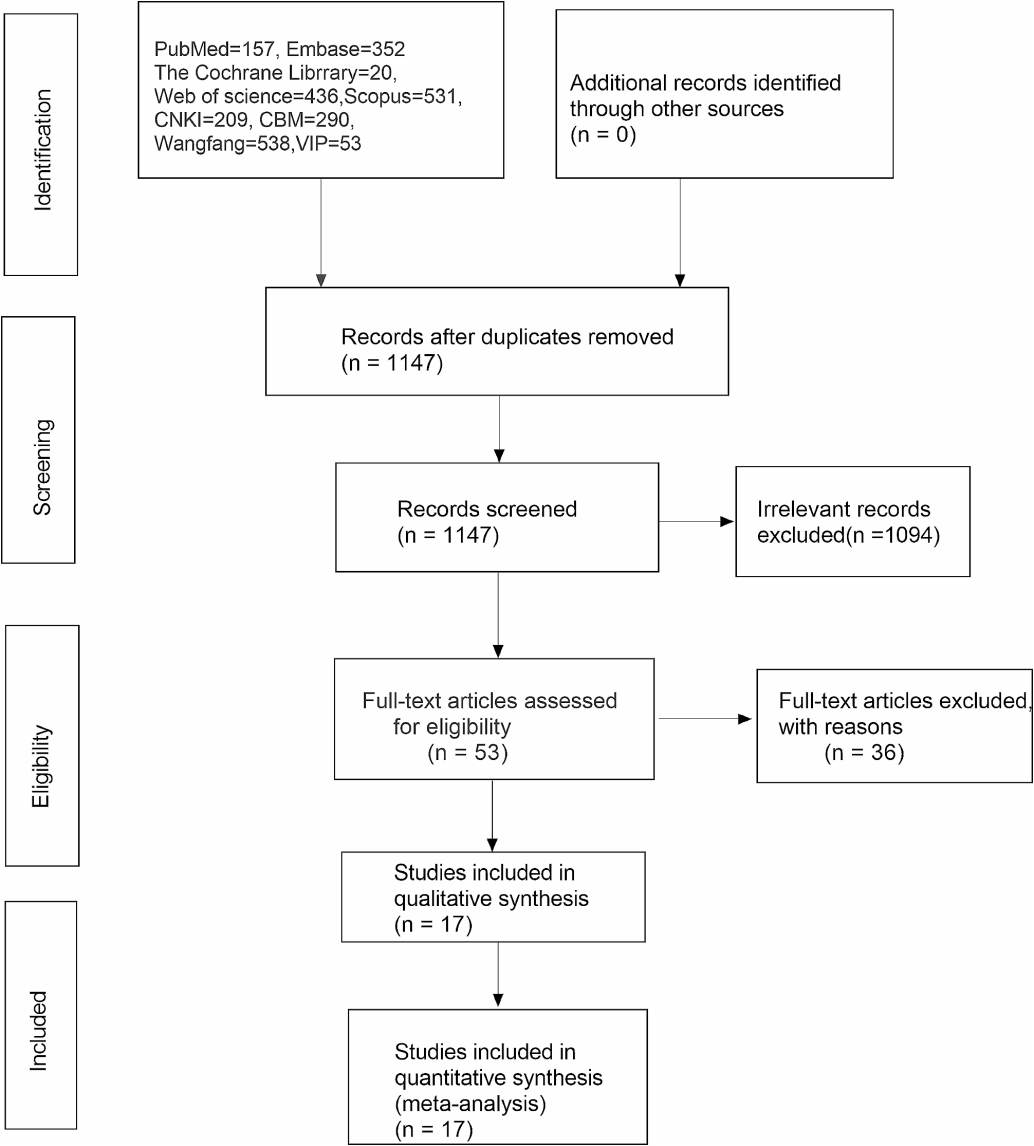
Flowchart of literature search of screening

**Fig. 3:**
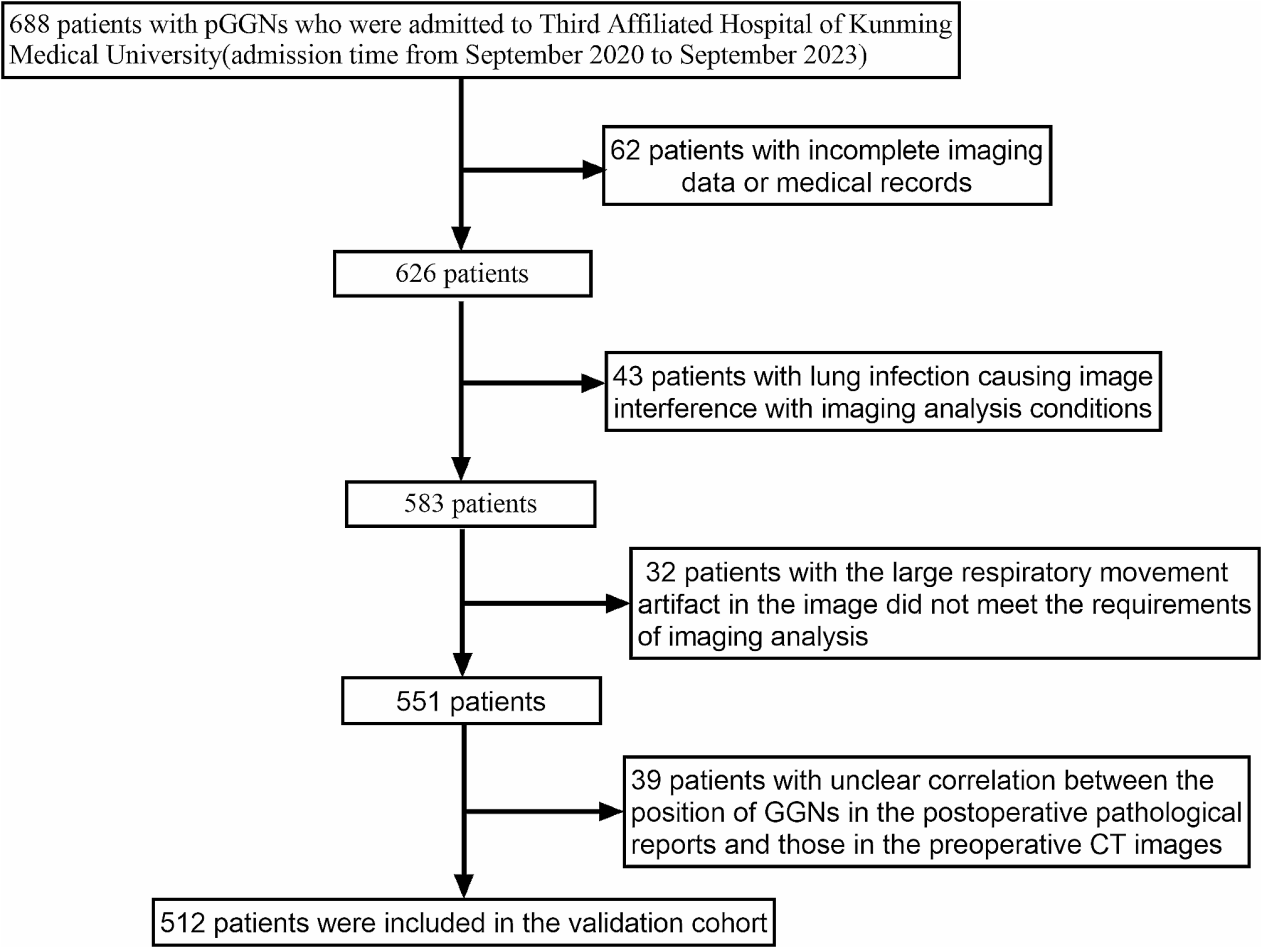
Process for the selection of patients in the validation cohort

CT scans were performed using a Siemens spiral CT scanner, featuring 64 rows and 128 slices. The scanning covered the entire lung, from the apex to the base. The technical parameters for the scan were set as follows: the tube voltage at 120 kV, the tube current at 100 mAs, a pitch setting of 1.0, and the slices had a thickness of 1 mm. In terms of reconstructing the images, a matrix of 512 × 512 was employed, along with an algorithm specifically designed for high-resolution lung imaging. Settings for the lung window were adjusted to a width ranging between 1200 and 1500 HU and a level between − 600 and − 700 HU. For the mediastinal window, settings were established with a width from 400 to 500 HU and a level set at 40 to 50 HU. These parameters were derived from plain CT scan images. Two experienced chest radiologists, each with over 15 years in the field, independently reviewed the images, blinded to the patients’ clinical and pathological data. Discrepancies in their evaluations were resolved through discussion. HRCT characteristics assessed included (1) the spiculation sign, characterized by spinous protrusions at the nodule edges, extending into the surrounding lung tissue(Fig. 1A); (2) pleural traction sign, identified by linear or tent-shaped shadows connecting the lesion to the pleura, sometimes presenting as a star-shaped shadow(Fig. 1B); (3) maximum diameter, measured on axial CT images [13]; and (4) mean CT value, calculated using a region of interest (ROI) cursor at the largest cross-section of the nodule, avoiding large bronchi, blood vessels, and any vacuoles/cavities [14].

##### Histopathological evaluation

In the training dataset, the classification of pGGNs was based on histopathological analysis of surgically removed specimens. These lesions were categorized into AIS, MIA, or IAC as per the 2021 classification criteria set by the World Health Organization [2]. For the diagnosis of these nodules, two seasoned pathologists, each with over 15 years of experience at the Pathology Department of the Third Affiliated Hospital of Kunming Medical University, conducted a joint review and confirmation.

### Statistical analysis

### Meta-analysis

Odds Radio(OR) and their 95% confidence interval(CI) were systematically compiled from the selected cohort studies. The identification of risk factors was based on the degree of variability across studies, determined using both the Q-test and the I² statistic. In cases where significant variability was observed, indicated by a P-value of less than 0.10 or an I² exceeding 50%, the synthesis of the pooled OR and 95% CI was conducted using a model that assumes random effects. Conversely, in the absence of notable heterogeneity, a fixed-effects model was employed. The robustness of the findings was assessed through sensitivity analyses, which involved the sequential omission of individual studies. The potential for publication bias within these studies was appraised utilizing Funnel plots and Egger tests and Begg test; a P-value greater than 0.05 was interpreted as a lack of substantial bias.

### Model development and validation

A mathematical model was developed using significant CT characteristics identified by meta-analysis, with each characteristic’s impact quantified by multiplying it with its corresponding regression coefficient (β), where β equals the natural logarithm of the OR. The imaging features of the patients were included at Third Affiliated Hospital of Kunming Medical University were analyzed. Continuous variables adhering to a normal distribution were presented as mean ± standard deviation, while those with skewed distributions were expressed as median (interquartile range). Frequency and proportion were used to represent categorical variables. The analysis of the ROC curve included the calculation of sensitivity and specificity, along with the area under the curve(AUC) and the ideal threshold. The AUC values, which vary between 0.5 and 1.0, are indicative of the model’s predictive precision, where larger numbers suggest enhanced accuracy. Calibration curves evaluated the model’s calibration, and decision curve analysis(DCA) assessed its clinical applicability.

## Results

### Description of the cohorts

#### Derivation cohort

In this meta-analysis, initial searches identified 2586 relevant publications, but ultimately only 17 studies were selected after excluding duplicates, irrelevant titles and abstracts, animal research, and articles requiring full-text review. These studies were all retrospective, comprising 7 in English and 10 in Chinese(Fig. 2). Quality assessment of these publications was conducted using the NOS, with 8 articles scoring 5 points, 7 articles scoring 6 points, and 2 articles scoring 7 points. All selected studies had NOS scores above 5, signifying their high quality, as illustrated in Table 1.

**Table 1:** Basic characteristics and quality evaluation results of the included literature

Author	Year	Country/	Sample size	Invasive	Pre-invasive	Outcomes	NOS scores
		Region					
Min Li et al. [15]	2022	China	119	66	53	(1)(9)	5
Yi-Lv Lv et al. [16]	2022	China	NA	NA	NA	(6)(7)(8)	5
Xinguan Yang et al. [17]	2021	China	150	95	55	(1)(2)(5)(7)(8)	5
Xuhong Min et al. [18]	2021	China	196	128	68	(1) (3)(4)(7)(9)	6
Fangqiu Fu et al. [19]	2021	China	432	331	101	(1)(2)(3)(4)	7
Lei Zhang et al. [20]	2020	China	170	92	78	(1)(5)(7)(8)(9)	6
Ye Yu et al. [21]	2020	China	148	98	50	(1)(4)(5) (7) (9)	6
Min Zheng et al. [22]	2019	China	163	102	61	(1) (4)	5
Ke yin et al. [23]	2019	China	200	124	76	(2)	5
Shuai Hu et al. [24]	2019	China	271	214	57	(1)(4)	6
Qian-Jun Zhou et al. [25]	2017	China	211	154	57	(1)(4)(6)(9)	6
Hongdou Ding et al. [26]	2017	China	361	275	86	(1)	5
Zhonggang Chen et al [27]	2021	China	189	157	32	(1)(8)	5
Jianyong Liao et al [28]	2020	China	81	38	43	(5)	5
Taichun Qiu et al [29]	2020	China	172	70	102	(2)(7)	6
Fuying Hu et al [11]	2021	China	344	211	133	(3)(5)(6)(8)(9)	7
Huan-Huan Yang et al [10]	2020	China	659	523	136	(3)(7)(8)	6

(1) maximum diameter; (2) regular shape; (3) pleura traction sign; (4) mean CT value (5) lobulation; (6) Vacuole sign; (7) air bronchogram sign; (8) speculation; (9) vascular convergence sign

#### Validation cohort

The study encompassed 512 individuals with pure ground glass nodules (Fig. 3), among whom 372 were female, representing 73% of the total cohort. Non-smokers comprised 412 of the participants, making up 81% of the group. Additionally, 410 patients were under the age of 60, accounting for 80% of the total (Table 2).

**Table 2:** The clinical and CT characteristic in the validation data set

	PIL (*n* = 289)	IAC (*n* = 223)
Sex/No.(%)		
Male	73(25.26)	67(30.04)
Female	216(74.74)	156(69.96)
Smoking/No.(%)		
Former/Current	59(20.42)	41(18.39)
Never	230(79.58)	182(81.61)
Age/No.(%)		
<60	228(78.89)	182(81.61)
≥ 60	61(21.11)	41(18.39)
CT feature		
Maximum diameter(cm)	1.0 (0.8, 1.3)	1.3 (1.1, 1.6)
Mean CT value(HU)	-630 (-700, -540)	-520 (-600, -460)
Spiculation sign(%)		
No	230 (79.58)	89 (39.91)
Yes	59 (20.42)	134 (60.09)
Pleura traction sign(%)		
No	260 (89.97)	167 (74.89)
Yes	29 (10.03)	56 (25.11)

### Model development and validation

**Fig. 4:**
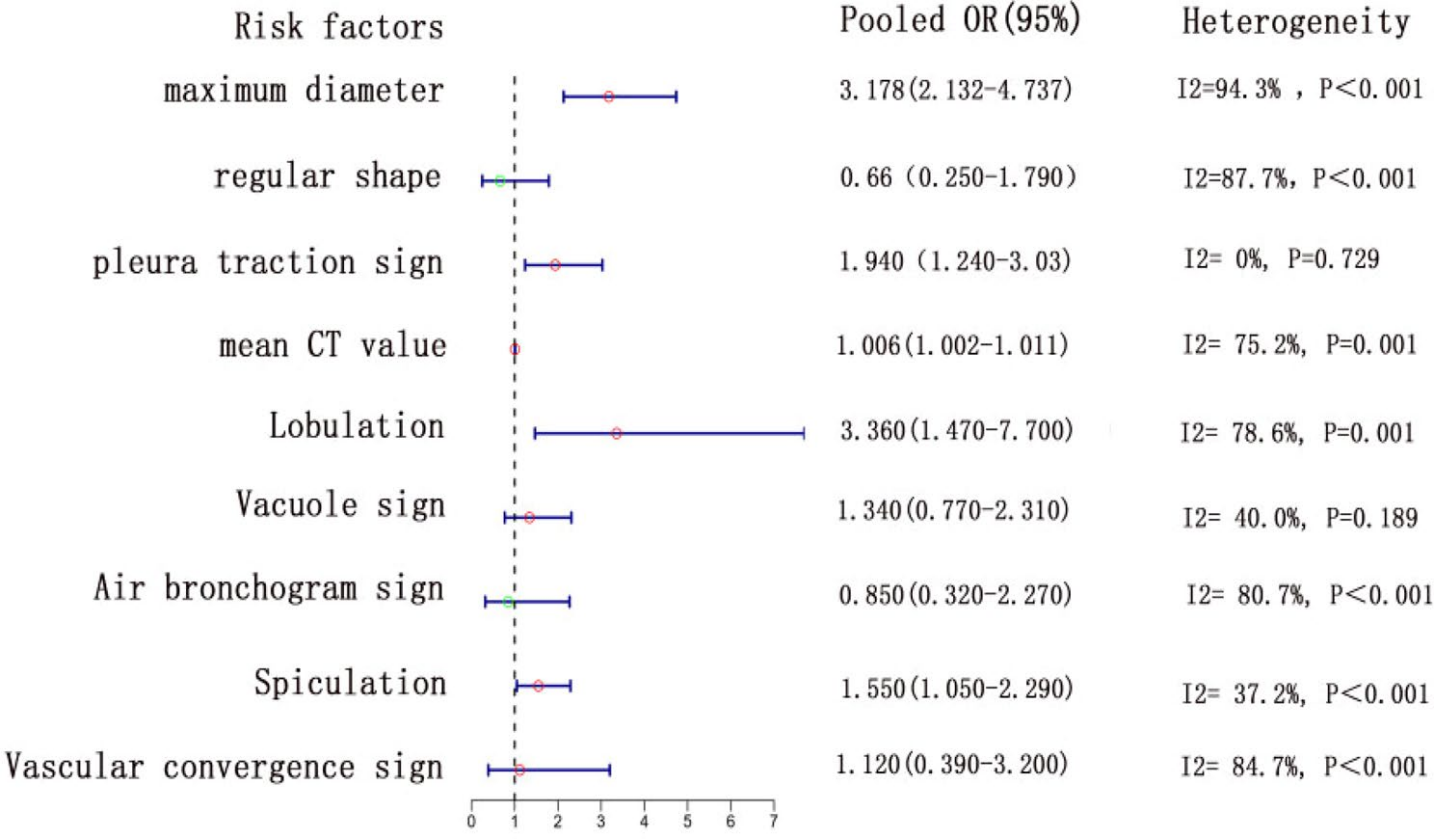
Pooled OR (95% CI) and heterogeneity test of the risk factors for IAC in pure ground-glass nodule

**Fig. 5:**
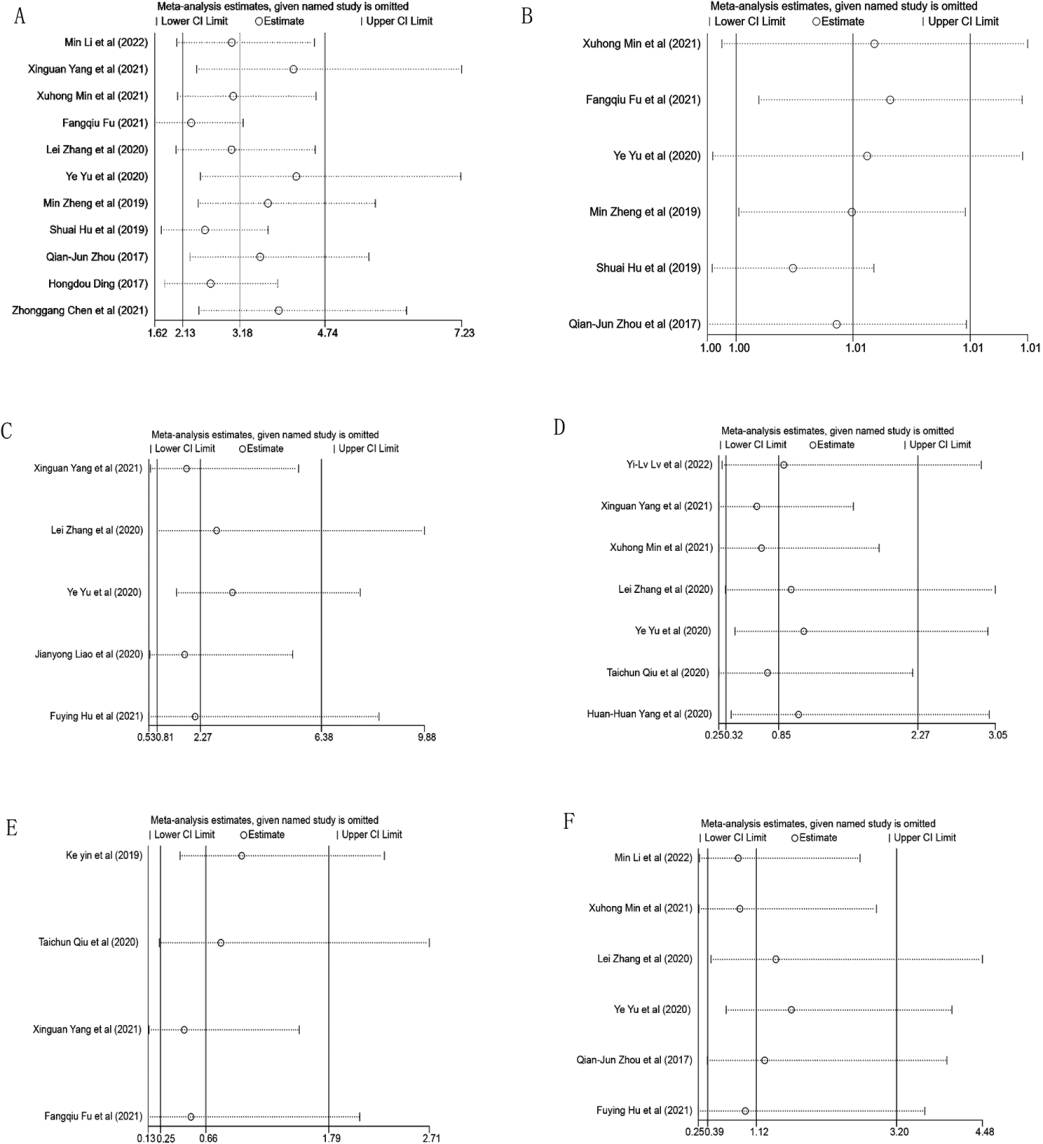
The sensitivity analysis of CT-based features for the included studies. **A**) maximum diameter; **B**) mean CT value; **C**) lobulation; **D**) air bronchogram sign; **E**) regular shape; **F**) vascular convergence sign

In the analyzed meta-study, four out of nine evaluated risk factors were linked to the onset of IAC. The study identified several factors with notable ORs: the largest tumor diameter (OR = 3.178, 95% CI: 2.132–4.737), average CT values (OR = 1.006, 95% CI: 1.002–1.011), pleural traction (OR = 1.94, 95% CI: 1.24–3.03) and spiculation (OR = 1.55, 95% CI: 1.05–2.29). Conversely, variables like vacuole and bronchial air signs, uniform tumor shape, lobulation, and vascular convergence showed no substantial correlation with IAC (*P* > 0.05), as depicted in Fig. 4. When subjected to sensitivity analysis, factors like maximum diameter, regular shape, average CT value, air bronchogram, and vascular convergence sign demonstrated consistency, whereas the lobulation sign exhibited variability as detailed in Fig. 5. Furthermore, The funnel plot shows no publication bias in the maximum diameter (provided in Supplementary Fig. 1) and Egger test and Begg test confirmed the absence of publication bias across all risk factors (*P* > 0.05).

**Fig. 6:**
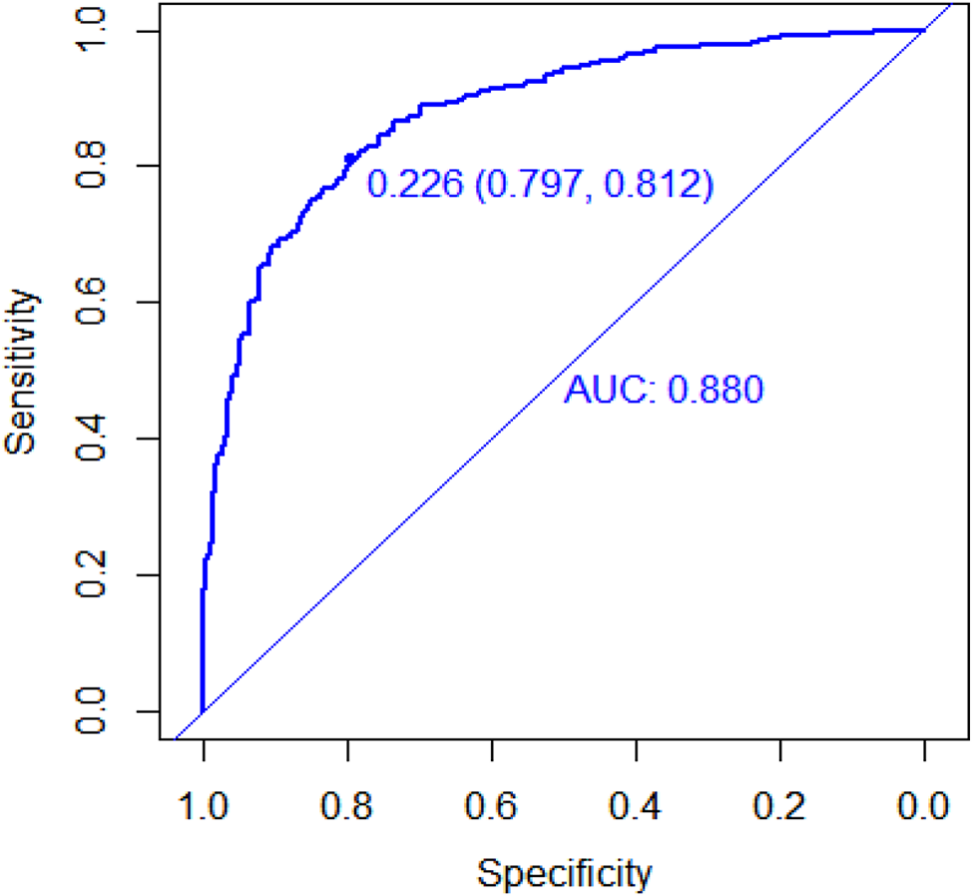
ROC curve of risk prediction model for IAC with pure ground-glass nodule in validation set

**Fig. 7:**
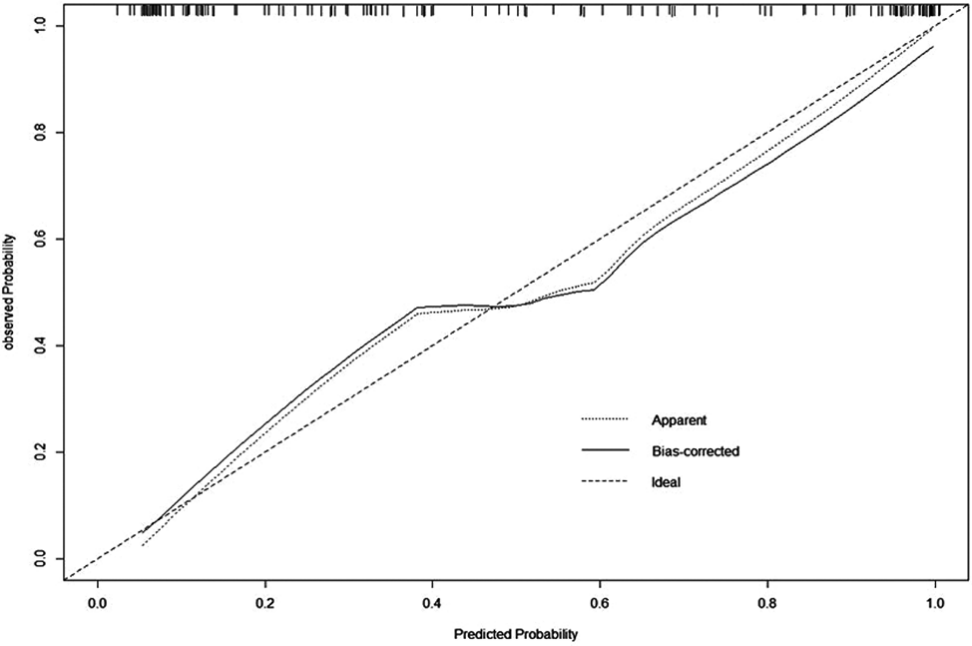
Calibration curve of risk prediction model for IAC with pure ground-glass nodule in validation set

**Fig. 8:**
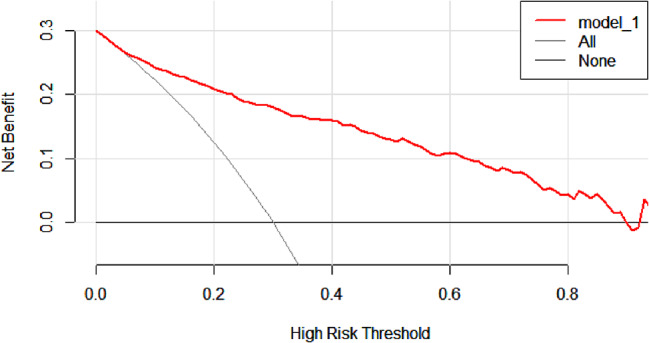
Decision analysis curve of risk prediction model for IAC with pure ground-glass nodule in validation set

The meta-analysis determined β values for several factors: 1.16 for maximum diameter, 0.01 for mean CT value, 0.66 for the presence of pleural traction, and 0.44 for spiculation sign. The derived mathematical formula is: 1.16×the largest lesion diameter + 0.01 × the average CT value + 0.66 × pleural traction + 0.44 × spiculation. Within the validation group, the ROC curve’s AUC was 0.88 (95% CI 0.794–0.868)(Fig. 6).The model exhibited an 81% sensitivity and an 80% specificity in predicting IAC. Furthermore, the analyses of the calibration curve (Fig. 7) and DCA (Fig. 8) for the validation group demonstrated the model’s accurate calibration and its clinical applicability.

## Discussion

The increasing use of HRCT has led to the detection of a higher number of ground glass nodules [30]. These nodules, often pure ground glass in nature, are typically slow-growing tumors, presenting challenges in their management [31]. Identifying invasive adenocarcinoma (IAC) in nodules that appear as pure ground glass is crucial, given the necessity for more comprehensive surgical approaches and timely intervention [6].

Recent research indicates that various CT imaging characteristics correlate with the development of IAC in pure ground glass nodules, though findings vary across studies [10, 19]. Meta-analysis, being the pinnacle of evidence-based medicine, offers more reliable predictive models than individual studies [32, 33]. This approach was utilized to amalgamate data from relevant cohort studies, identifying IAC risk factors and formulating a predictive model. This model, with an AUC of 0.88, demonstrates 81% sensitivity and 80% specificity in predicting IAC, showcasing high calibration accuracy and clinical utility. Such findings are instrumental in guiding the timing and approach of surgical interventions for pure ground glass nodules.

In this meta-analysis, We extended the search time again.17 articles were reviewed, identifying four IAC risk factors: maximum diameter, mean CT value, pleural traction sign, and spiculation sign. Compared to previous study [34]. we re-extracted the OR of the maximum diameter and average CT values in each study to obtain a new forest map of combined results((provided in Supplementary Figs. 2, 3). It was noted that IAC risk escalates with an increase in the maximum diameter of pure ground glass nodules, possibly linked to heightened tumor aggressiveness and structural changes. A higher mean CT value, observed in IAC cases, might result from the myofibroblast matrix thickening due to tumor cell invasion into normal pulmonary tissue. Contrarily, Fu et al [19] reported that mean CT value was not a reliable identifier of IAC, possibly attributed to minimal group differences stemming from sample selection. The spiculation sign, indicative of tumor cell proliferation and local infiltration, increases with tumor invasion, aligning with our meta-analysis findings. Several studies have indicated no correlation between the pleural depression sign and IAC [15, 35], but their limited sample sizes may affect these conclusions. Our meta-analysis, however, found an independent association between IAC and the pleural traction sign.

We re-performed sensitivity analysis for all risk factors exhibiting significant heterogeneity. Results remained stable for all except lobulation, which demonstrated instability upon sensitivity analysis. By omitting the study by Yu et al [21], a statistically significant difference was observed in the incidence of lobulation between IAC and PIL in pGGNs. This variation could be attributed to disparities in clinical features or patient demographics. In addition, publication bias for all CT features was reassessed, which made our results more robust.

In our validation cohort, the prevalence of pure ground glass nodules was notably higher among women, non-smokers, and individuals under 60 years, aligning with other researchers observations [36, 37]. The reasons behind this pattern remain uncertain and warrant further investigation.

Previous study [34] have identified risk factors for differentiating PIL from IAC in pGGNs. However, Dai et al [38] found that using a single CT feature qualitatively has limited diagnostic value for differentiating PIL from IAC in pGGNs. A combination of both quantitative and qualitative CT features has shown effective in determining the invasiveness of pGGNs. Predictive models for IAC in pure ground glass nodules have been developed by several researchers. Hu et al. [11] suggested a model integrating multiple imaging characteristics for predicting invasiveness. Similarly, Min et al. [18] and Liu et al [39] emphasized the efficacy of combining quantitative and qualitative features in their diagnostic models. Yet, these models, primarily based on small, single-cohort studies, lack comprehensive validation, reducing their clinical applicability. Based on the high-level evidence of meta-analysis, we for the first time use regression coefficient combined with the results of meta-analysis to establish a prediction model. For validation, 512 patients with pure ground glass nodules from The Third Affiliated Hospital of Kunming Medical University were included. This is a large sample validation compared to previous studies, making the results more robust. Compared to previous studies [22, 26], our model shows a better predictive performance, with an AUC of 0.88 (95% CI: 0.852–0.909), with 81% sensitivity and 80% specificity. Additionally, calibration curves and decision analysis were conducted, yielding favorable results. This model can effectively identify IAC in pure ground glass nodules, thus aiding treatment decisions.

Our study has certain limitations. Firstly, due to variations in study design and CT scan parameters, heterogeneity across the literature is unavoidable. Secondly, the inclusion of only retrospective studies might introduce selection bias. Thirdly, as all studies were conducted in China, this may restrict the broader applicability of our findings. Lastly, the validation cohort was derived from a single center, limiting the model’s predictive efficacy testing. Multi-center patient testing is necessary for further validation.

## Conclusion

The meta-analysis conducted led to the creation of a reliable and effective predictive model for assessing the risk of IAC in cases of pure ground glass nodules. This model integrates several key parameters, including the largest diameter, average CT values, indications of spiculation, and signs of pleural traction. It offers substantial utility in assisting clinicians in making informed decisions about the treatment of pure ground glass nodules.

### Electronic supplementary material

Below is the link to the electronic supplementary material.

Supplementary Material 1

Supplementary Material 2

Supplementary Material 3

## Data Availability

The datasets generated and/or analyzed during the current study are not publicly available due sharing data is not included in our research institution review board but are available from the corresponding author on reasonable request.

## References

[1] Aberle DR, Adams AM, Berg CD (2011). Reduced lung-cancer mortality with low-dose computed tomographic screening. N Engl J Med.

[2] Li Y, Xie HK, Wu CY. Interpretation of lung tumours in the WHO classification of thoracic tumours (5th edition). China Oncol. 2021;31(7):574e580.

[3] Naidich DP, Bankier AA, MacMahon H (2013). Recommendations for the management of subsolid pulmonary nodules detected at CT: a statement from the Fleischner Society. Radiology.

[4] Lee GD, Park CH, Park HS (2019). Lung adenocarcinoma invasiveness risk in pure ground-glass opacity lung nodules smaller than 2 cm. Thorac Cardiovasc Surg.

[5] Yotsukura M, Asamura H, Motoi N (2021). Long-term prognosis of patients with resected Adenocarcinoma in situ and minimally invasive adenocarcinoma of the lung. J Thorac Oncol.

[6] Saji H, Okada M, Tsuboi M (2022). Segmentectomy versus lobectomy in small-sized peripheral non-small-cell lung cancer (JCOG0802/WJOG4607L): a multicentre, open-label, phase 3, randomised, controlled, non-inferiority trial. Lancet.

[7] Jiang GN, Chen C, Zhu YM (2018). Shanghai pulmonary hospital experts consensus on the management of ground-glass nodules suspected as lung adenocarcinoma (version 1). Chin J Lung Cancer.

[8] Xinli W, Xiaoshuang S, Chengxin Y, Qiang Z. CT-Assisted Improvements in the Accuracy of the Intraoperative Frozen Section Examination of Ground-Glass Density Nodules. Comput Math Methods Med. 2022. 2022: 8967643. 10.1155/2022/8967643 PMC8759914 35035526

[9] Whybra P, Zwanenburg A, Andrearczyk V (2024). The image Biomarker Standardization Initiative: standardized Convolutional filters for reproducible Radiomics and enhanced clinical insights. Radiology.

[10] Yang HH, Lv YL, Fan XH (2020). Factors distinguishing invasive from pre-invasive adenocarcinoma presenting as pure ground glass pulmonary nodules. Radiat Oncol.

[11] Hu F, Huang H, Jiang Y (2021). Discriminating invasive adenocarcinoma among lung pure ground-glass nodules: a multi-parameter prediction model. J Thorac Dis.

[12] Wang Z, Zhu W, Lu Z, Li W, Shi J (2021). Invasive adenocarcinoma manifesting as pure ground glass nodule with different size: radiological characteristics differ while prognosis remains the same. Transl Cancer Res.

[13] Si MJ, Tao XF, Du GY (2016). Thin-section computed tomography-histopathologic comparisons of pulmonary focal interstitial fibrosis, atypical adenomatous hyperplasia, adenocarcinoma in situ, and minimally invasive adenocarcinoma with pure ground-glass opacity. Eur J Radiol.

[14] Travis WD, Brambilla E, Noguchi M (2011). International association for the study of lung cancer/american thoracic society/european respiratory society international multidisciplinary classification of lung adenocarcinoma. J Thorac Oncol.

[15] Li M, Wang YF, Jiang WZ (2022). The value of qualitative and quantitative parameters of dual-layer spectral detector CT plain scan in predicting the invasiveness of pure ground-glass pulmonary nodules. Chin J Radiol.

[16] Lv YL, Zhang J, Xu K (2022). Computed tomography versus frozen sections for distinguishing lung adenocarcinoma: a cohort study of concordance rate. Asian J Surg.

[17] Yang XG, Li X, Tong QY (2021). Efficiency of preoperative thin-section CT parameters for predicting invasive growth of pure ground-glass nodules in lung adenocarcinoma. Guangxi Med J.

[18] Min XH, Song QL, Yu YQ (2021). The clinical value of the logistic regression model with a combination of three-dimensional CT quantitative and qualitative parameters in predicting the invasiveness of pure ground glass nodules. Chin J Radiol.

[19] Fu F, Zhang Y, Wang S (2021). Computed tomography density is not associated with pathological tumor invasion for pure ground-glass nodules. J Thorac Cardiovasc Surg.

[20] Zhang l, Xie XD, Shen WR (2020). The value of CT features in predicting the invasiveness of PGGN. J Pract Radiol.

[21] Yu Y, Zhang Y, Zhang F (2020). Value of CT signs in determining the invasiveness of lung adenocarcinoma manifesting as pGGN. Int J Med Radiol.

[22] Zheng M, Huang XM (2019). Evaluation the invasion of pure ground-glass nodules in lung based on CT quantitative parameters. Chin J CT MRI.

[23] Yin K, Wu JL, Qiu TC (2019). Establishment of a model for the diagnosis of invasive adenocarcinoma of the lung with high-resolution CT signs. Chin J Med Image.

[24] Hu S, Ge Y, Li MY (2019). Quantitative assessment of invasive pulmonary adenocarcinoma as pure ground glass nodule using thin-slice CT. J Pract Radiol.

[25] Zhou QJ, Zheng ZC, Zhu YQ (2017). Tumor invasiveness defined by IASLC/ATS/ERS classification of ground-glass nodules can be predicted by quantitative CT parameters. J Thorac Dis.

[26] Ding H, Shi J, Zhou X (2017). Value of CT characteristics in Predicting Invasiveness of Adenocarcinoma presented as Pulmonary Ground-Glass nodules. Thorac Cardiovasc Surg.

[27] Chen ZG, Xia TY, Fu GZ (2021). Value of CT scanning in predicting the infiltration of subpleural pure ground glass nodules in patients with lung adenocarcinomas. Jiangsu Med J.

[28] Liao JY, Du JB, Liu YX (2020). Influencing factors of invasive adenocarcinoma in patients with pulmonary pure ground glass nodules on CT. Med J Chin PAP.

[29] Qiu T, Ru X, Yin K, Yu J, Song Y, Wu J (2020). Two nomograms based on CT features to predict tumor invasiveness of pulmonary adenocarcinoma and growth in pure GGN: a retrospective analysis. Jpn J Radiol.

[30] Wiener RS, Gould MK, Arenberg DA (2015). An official American Thoracic Society/American College of Chest Physicians policy statement: implementation of low-dose computed tomography lung cancer screening programs in clinical practice. Am J Respir Crit Care Med.

[31] Takahashi S, Tanaka N, Okimoto T (2012). Long term follow-up for small pure ground-glass nodules: implications of determining an optimum follow-up period and high-resolution CT findings to predict the growth of nodules. Jpn J Radiol.

[32] Pek S, Lim SC, Ang K (2020). Endothelin-1 predicts incident diabetic peripheral neuropathy in type 2 diabetes: a cohort study. Eur J Endocrinol.

[33] Al Sayah F, Soprovich A, Qiu W, Edwards AL, Johnson JA (2015). Diabetic Foot Disease, Self-Care and clinical monitoring in adults with type 2 diabetes: the Alberta’s caring for diabetes (ABCD) cohort study. Can J Diabetes.

[34] Yang Y, Xu J, Wang W (2023). Meta-analysis of the correlation between CT-based features and invasive properties of pure ground-glass nodules. Asian J Surg.

[35] Ikehara M, Saito H, Kondo T (2012). Comparison of thin-section CT and pathological findings in small solid-density type pulmonary adenocarcinoma: prognostic factors from CT findings. Eur J Radiol.

[36] Zhang Y, Jheon S, Li H (2020). Results of low-dose computed tomography as a regular health examination among Chinese hospital employees. J Thorac Cardiovasc Surg.

[37] Luo X, Zheng S, Liu Q (2017). Should nonsmokers be excluded from early Lung Cancer screening with low-dose spiral computed tomography? Community-based practice in Shanghai. Transl Oncol.

[38] Dai J, Yu G, Yu J (2018). Can CT imaging features of ground-glass opacity predict invasiveness? A meta-analysis. Thorac Cancer.

[39] Liu J, Yang X, Li Y (2022). Development and validation of qualitative and quantitative models to predict invasiveness of lung adenocarcinomas manifesting as pure ground-glass nodules based on low-dose computed tomography during lung cancer screening. Quant Imaging Med Surg.

