# Supplementary material for: Development and validation of a risk prediction model for invasiveness of pure ground-glass nodules based on a systematic review and meta-analysis
Source: BMC Med Imaging. 2024 Jun 17;24:149. doi: 10.1186/s12880-024-01313-5 (PMC11184730; doi:10.1186/s12880-024-01313-5)
Supplement: Supplementary file 2 — Supplementary Material 2 [file 12880_2024_1313_MOESM2_ESM.docx]

Figure 2 Combined forest map with maximum diameters
